# Progression of medial meniscal extrusion following anterior cruciate ligament reconstruction correlates with graft diameter: a retrospective longitudinal study

**DOI:** 10.1186/s12891-025-08950-z

**Published:** 2025-07-18

**Authors:** Shinnosuke Hada, Jun Tomura, Youngji Kim, Keiichi Yoshida, Haruka Kaneko, Yoshitomo Saita, Mitsuaki Kubota, Yuji Takazawa, Muneaki Ishijima

**Affiliations:** 1 Department of Orthopaedic Surgery, Juntendo University School of Medicine, Tokyo, Japan; 2 Department of Orthopaedic Surgery, Juntendo Tokyo Koto Geriatric Medical Center, Tokyo, Japan

**Keywords:** Medial meniscus extrusion, Anterior cruciate ligament, Posttraumatic osteoarthritis, Graft diameter

## Abstract

**Background:**

Posttraumatic osteoarthritis (PTOA), a subtype of knee osteoarthritis (OA), develops following intra-articular injuries such as fractures, ligament tears, or meniscal damage, accounting for approximately 12% of symptomatic knee OA. Notably, 87% of patients with anterior cruciate ligament (ACL) injuries are expected to develop PTOA, and there is currently no established method to prevent its progression. While ACL reconstruction (ACL-R) restores joint stability, it does not necessarily halt PTOA development. Previous MRI-based studies have identified medial meniscal extrusion (MME) and medial tibial osteophyte (MTO) formation as early indicators of PTOA. Moreover, ACL-R can trigger intra-articular hematoma and inflammation, especially due to the drilling of bone tunnels for graft insertion. Larger graft diameters may cause more joint trauma; however, their association with early PTOA changes remains unclear. This study aimed to investigate the relationship between graft diameter and early PTOA progression as observed on MRI.

**Methods:**

This retrospective cohort study included 42 patients (30 males, 12 females) who underwent arthroscopic ACL-R by a single surgeon. MRI scans were obtained preoperatively and once postoperatively, between 5 and 12 months after surgery. Graft diameter was measured at both femoral and tibial tunnel sites using a cylindrical sizer, and the average value was used for analysis. PTOA changes were assessed based on progression of MME and osteophyte formation. Patients were categorized into larger and smaller graft groups using the cohort’s median graft diameter (9.25 mm) as the cutoff.

**Results:**

Graft diameter significantly correlated with MME progression (ΔMME: *r* = 0.48, *p* = 0.001) and MTO progression (ΔMTO: *r* = 0.31, *p* = 0.048). ΔMME also strongly correlated with ΔMTO (*r* = 0.67, *p* < 0.001), but not with medial femoral osteophyte (ΔMFO: *r* = 0.23, *p* = 0.15) or lateral compartment changes. The larger graft group exhibited significantly greater ΔMME (*p* = 0.005) and ΔMTO (*p* = 0.03) compared to the smaller graft group.

**Conclusion:**

Larger graft diameters were associated with greater early progression of MME and MTO following ACL-R. These findings suggest a possible mechanical or inflammatory contribution to early PTOA development after reconstruction.

## Background

Although knee osteoarthritis (OA) is the most common cause of knee pain and movement disorders, no established treatment prevents its progression. This is partly because knee OA has various subtypes, each requiring a clearer understanding to develop treatment interventions [1]. Posttraumatic OA (PTOA), a subtype that develops after intra-articular fractures, ligament injuries, cartilage damage, or meniscus injuries, accounts for approximately 12% of symptomatic knee OA [2]. Notably, 87% of patients with anterior cruciate ligament (ACL) injuries eventually develop PTOA [3, 4]. Residual knee instability after ACL injury contributes to secondary meniscus and cartilage damage, increasing PTOA risk. Consequently, ACL reconstruction (ACL-R) with autologous tendon grafts is a common choice for younger and highly active patients. However, long-term studies have shown that ACL-R does not prevent PTOA progression [5].

Although many studies have used long-term radiographic evaluations to assess PTOA after ACL-R [3], magnetic resonance imaging (MRI) is vital for identifying early-stage pathology and potential treatment targets to slow disease progression [6]. In recent years, MRI-based pathological research has highlighted medial meniscal extrusion (MME) as an early OA indicator. MME occurs when the medial meniscus is extruded in an extra-articular direction, increasing mechanical stress on the medial articular cartilage, which accelerates degeneration and markedly raises OA risk [7–9].

There is a growing interest in early PTOA treatments targeting MME [10], although MME’s pathogenesis, especially its causes, remains unclear. A longitudinal study comparing preoperative MRI findings with early postoperative PTOA markers (average 7.6 months after ACL-R surgery) found no notable progression of cartilage or meniscus damage, whereas MME incidence increased and was strongly correlated with medial tibial osteophyte (MTO) formation. This suggests that MME may result from the medial meniscus being displaced by the MTO [4, 11].

Osteophytes have traditionally been considered a late-stage OA feature [12], but studies using OA model mice have revealed that osteophyte formation begins before OA cartilage lesions appear [13, 14]. Furthermore, MRI-based research has confirmed that osteophytes frequently develop in patients with early-stage OA [15] and even in healthy middle-aged and elderly individuals [16], often preceding cartilage and meniscus damage. Thus, osteophytes, akin to MME, are valuable markers of early PTOA.

Although ACL-R improves joint instability and reduces secondary meniscus and cartilage damage, it does not inhibit PTOA progression. This may be attributed to joint hematoma and prolonged intra-articular inflammation following surgery due to joint invasion, with levels of knee OA markers in the joint fluid remaining elevated for long periods, even for over 5 years [17]. The most invasive aspect of ACL-R surgery is drilling a bone tunnel for graft insertion, which in animal models promotes cartilage damage and osteophyte formation as well as the release of transforming growth factor-beta (TGF-β) and matrix metalloproteinase-13 (MMP13) [18]. A larger graft diameter necessitates a larger bone tunnel, causing greater joint damage. However, the relationship between graft diameter and early PTOA lesions remains poorly understood.

Therefore, the aim of this study was to investigate the association between graft diameter and early MRI-detected PTOA features, particularly MME and osteophyte formation, following ACL-R. We hypothesized that larger graft diameters would be associated with increased MME and osteophyte progression in the early postoperative period.

## Materials and methods

### Ethics

This retrospective, cross-sectional, observational study was conducted between May 2012 and January 2025. The study protocol complied with the principles of the Declaration of Helsinki and was approved by the Ethical Review Board Committee of our university (approval number: E22-0166). As this was a retrospective study using anonymized data, the committee waived the requirement for patient informed consent.

### Subjects

This study included patients who underwent primary arthroscopic ACL-R surgery using hamstring tendon autografts, performed by a single experienced surgeon. Eligible patients were required to have complete MRI data available both preoperatively and postoperatively. Exclusion criteria comprised the use of grafts other than hamstring tendons, revision ACL-R, concomitant injuries to other knee ligaments, severe cartilage lesions, and incomplete or missing MRI data. After applying these criteria, a total of 42 patients were included in the analysis (30 males and 12 females). As this was a retrospective study, a priori sample size calculation was not performed. We included all eligible patients who met the inclusion criteria during the study period.

### Surgical treatment

Arthroscopic-assisted anatomical single-bundle ACL-R was performed using a semitendinosus tendon autograft. The semitendinosus tendon was prepared as a five-strand hamstring graft, measuring 50 mm in total length after looping. To determine the drill diameter for bone tunnel drilling, the graft diameter was measured on the femoral and tibial sides using a cylindrical measuring instrument, and the average of these measurements was recorded as the graft diameter for this study. The graft was fixed proximally to the lateral femoral cortex and distally to the medial tibia. Postoperatively, there were no restrictions on weight-bearing or range of motion, and no orthotic devices were used [4, 19]. Postoperative tunnel positions were evaluated using the quadrant method for the femoral tunnel and anteroposterior and mediolateral ratios for the tibial tunnel, in accordance with previously established criteria [20, 21].

### Physical and activity assessments

Knee joint stability was assessed at follow-up using the pivot shift test, graded on a 0–3 scale. Additionally, activity levels before and after surgery were evaluated using the Tegnar activity scale [22].

## X-ray assessments

X-ray evaluation was performed using the Kellgren–Lawrence (K/L) grade [23] to assess OA severity, along with measurements of the femorotibial angle and medial joint space width. The medial joint space width was determined at the center point of the medial femoro-tibial compartment on a radiograph using a 0.1-mm graduated magnifying lens. Radiographs were taken in standing, extension, anterior–posterior, and lateral views [24].

### MRI evaluation

All patients underwent two MRI scans: a preoperative scan (first), which was performed within 3 months before surgery for diagnostic purposes, and a postoperative scan (second), which was performed between 5 and 12 months after surgery. Both scans were performed in the supine position without weight-bearing. Imaging sequences included proton density-weighted images in the coronal and sagittal planes, along with proton-weighted fat-suppressed images. An experienced orthopedic surgeon (S.H.), specializing in orthopedic MRI for over 15 years, evaluated the images [4].

To assess early PTOA-related morphological changes, including osteophytes, cartilage damage, bone marrow lesions, meniscus tears, and extrusion, MRI findings were scored using the simplified modified anterior cruciate ligament osteoarthritis score (ACLOAS) system [6, 25]. A longitudinal evaluation was performed to determine whether scores for the medial tibiofemoral joint (MTFJ), lateral tibiofemoral joint (LTFJ), and patellofemoral joint (PFJ) improved or worsened before and after ACL-R.

### Bone marrow lesions

Bone marrow lesions were classified as follows: grade 0: no lesion; grade 1: lesions occupying < 33% of the subregional volume; grade 2: lesions occupying 33–66% of the subregional volume;

grade 3: lesions occupying > 66% of the subregional volume.

### Cartilage

Articular cartilage integrity was assessed on proton density-weighted images and classified as follows: grade 0: no cartilage deficit; grade 1: cartilage deficit > 10% of the cartilage surface; grade 2: cartilage deficit 10–75% of the cartilage surface; grade 3: cartilage deficit > 75% of the cartilage surface.

### Osteophytes

Osteophytes are cartilage-covered bony projections that develop at the margins of the knee joint. They were classified into four grades: grade 0: none; grade 1: small; grade 2: medium; grade 3: large.

### Meniscal tears

Meniscal integrity was categorized into four grades: grade 0: normal; grade 1: intrameniscal hyperintensity not reaching the surface; grade 2: meniscal tear; grade 3: meniscal maceration.

### Meniscal extrusion

MME and lateral meniscal extrusion (LME) were scored on a mid-coronal MRI slice at the level of the largest medial tibial spine relative to the tibial plateau edge, using the following grading system: grade 0: no extrusion; grade 1: extrusion < 50% of the meniscal coronal length; grade 2: extrusion ≥ 50% of the meniscal coronal length.

### Quantitative measurement of meniscal extrusion and osteophyte widths

**Fig. 1:**
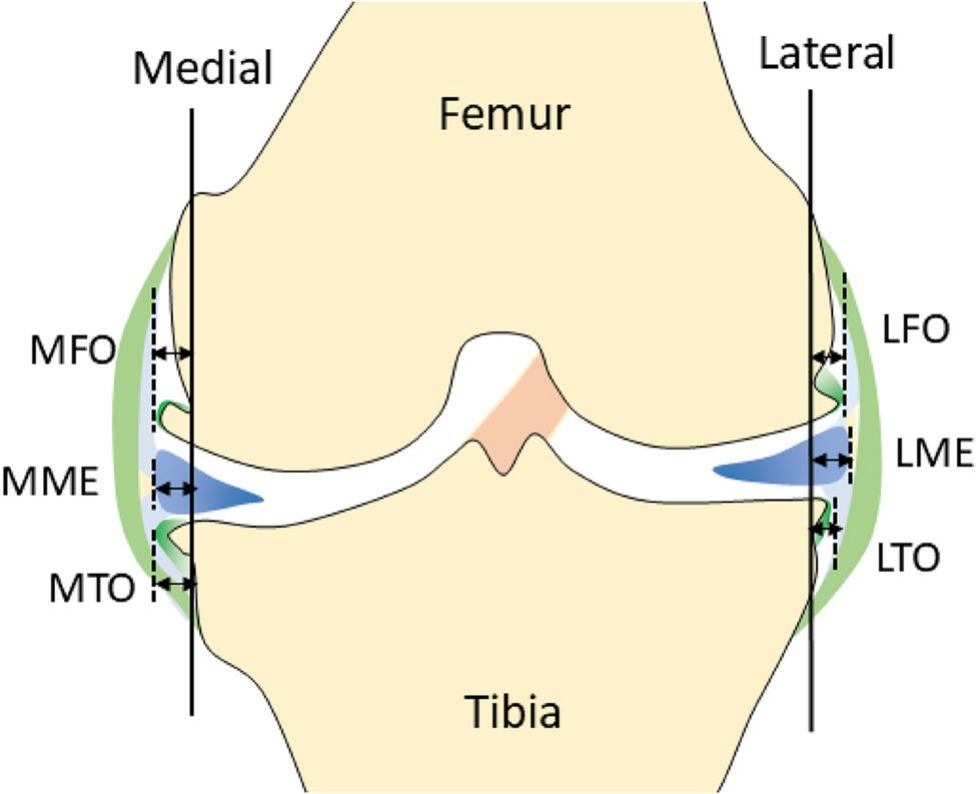
Schematic representation of meniscal extrusion and osteophyte width measurements. Schematic illustration depicting the measurement methods for medial meniscal extrusion (MME) and lateral meniscal extrusion (LME). Extrusion distance was defined as the distance from the outermost edge of the meniscus to a reference line connecting the femoral and tibial cortices, excluding osteophytes. Dotted lines differentiate the cartilage and bone components of osteophytes. Line between the bony part of the osteophyte and the tibial cortex was delineated by tracing the image. MFO, medial femoral osteophyte; MTO, medial tibial osteophyte; LFO, lateral femoral osteophyte; LTO, lateral tibial osteophyte

To allow for a more detailed analysis, meniscal extrusion width and osteophyte width were quantitatively measured. MME and LME were measured as the distance from the outermost meniscal edge to the line connecting the femoral and tibial cortices. Cortical bone was defined as the portion excluding osteophytes. Osteophyte width was measured, including the cartilage at the osteophyte tip, for the MTO, medial femoral osteophyte (MFO), lateral tibial osteophyte (LTO), and lateral femoral osteophyte (LFO) [4, 15] (Fig. 1).

### Reproducibility measurements

To assess interobserver reproducibility, two independent observers (S.H. and J.T.) performed the MRI evaluations while blinded to patient information. The intraobserver reproducibility of the modified ACLOAS assessment, MME width, and osteophyte width was measured using the interclass correlation coefficient (ICC), with the following results obtained: modified ACLOAS: ICC = 0.94 [95% confidence interval (CI): 0.91–0.96]; MME width: ICC = 0.95 (95% CI: 0.78–0.98); osteophyte width: ICC = 0.94 (95% CI: 0.85–0.98). Interobserver reproducibility was also high: modified ACLOAS: ICC = 0.90 (95% CI: 0.86–0.93); MME width: ICC = 0.93 (95% CI: 0.76–0.98); osteophyte width: ICC = 0.94 (95% CI: 0.84–0.97).

### Statistical analysis

Longitudinal changes in preoperative and postoperative MRI findings were analyzed in relation to graft diameter. All statistical analyses were conducted using SPSS 21.0 (SPSS Institute, Chicago, IL, USA). Changes in modified ACLOAS, osteophyte width, and meniscal extrusion width were compared using the paired *t*-test. Correlations between the longitudinal changes (Δ) in osteophyte and meniscal extrusion widths and graft diameter were evaluated using the Spearman correlation coefficient and independent *t*-test between the two groups. *P* < 0.05 was considered statistically significant. In the comparison between two groups based on graft diameter, patients were classified into a smaller-diameter group and a larger-diameter group using the median value within our cohort (9.25 mm) as the threshold, since no clear reference value has been established in the literature.

## Results

### Patient characteristics

The 42 patients with ACL injuries had an average age of 28.8 (± 9.4) years, and their Tegnar activity scale scores were 7.2 (± 1.8) preoperatively and postoperatively. Among these patients, 29 had K/L grade 0, 12 had grade 1, and 1 had grade 2 on radiograph evaluations. The average femorotibial angle and medial joint space was 176.6° (± 2.3°) and 4.0 (± 0.4) mm, respectively. The average graft diameter was 9.3 (± 0.9) mm, with 10 mm being the most common diameter (Table 1). During arthroscopic evaluation at the time of ACL reconstruction, medial meniscal injury was identified in 5 patients and lateral meniscal injury in 2 patients. These were treated with either partial meniscectomy or suture repair, depending on the tear pattern and stability. No cartilage lesions greater than International Cartilage Regeneration & Joint Preservation Society Grade 1 [4] were observed. The positions of both femoral and tibial tunnels were found to be generally within the acceptable anatomical ranges based on prior literature [20, 21].The details of the measurements are presented in Table 1. Additionally, correlation analysis revealed no significant association between tunnel positions and the progression of MME or LME. Specifically, for MME, the femoral tunnel height (*r* = −0.23, *p* = 0.13) and depth (*r* = −0.04, *p* = 0.76), as well as the tibial tunnel anteroposterior (*r* = −0.14, *p* = 0.37) and mediolateral (*r* = 0.02, *p* = 0.92) positions, showed no significant correlations. Similarly, for LME, the femoral tunnel height (*r* = 0.002, *p* = 0.99) and depth (*r* = 0.17, *p* = 0.28), and the tibial tunnel anteroposterior (*r* = 0.16, *p* = 0.30) and mediolateral (*r* = −0.15, *p* = 0.32) positions were also not significantly correlated with extrusion progression.

**Table 1:** Patient characteristics

Patient characteristics	
n	42
Age	28.9 (9.3)
Gender (male/female)	30 (71.4%)/12 (28.6%)
BMI	25.0 (3.6)
TAS (preoperation)	7.2 (1.8)
TAS (postoperation)	7.2 (1.8)
Pivot shift grade (postoperation)	Grade 0:19; Grade 1:23
X-ray	
K/L grade (0/1/2)	29/12/1
Femorotibial angle (°)	176.6 (2.3)
Medial joint space width (mm)	4.0 (0.4)
Tunnel positions	
Femoral tunnel position: Depth	25.0% (4.2)
Femoral tunnel position: Height	30.9% (3.7)
Tibial tunnel position: A-P direction	40.4% (4.1)
Tibial tunnel position: M-L direction	43.7% (2.5)
Graft diameter	
Average (mm)	9.3 (± 0.9)
7.5	2
8.0	5
8.5	4
9.0	8
9.5	8
10.0	11
10.5	3
11.0	1

Data are presented as means (with standard deviations). *BMI* Body mass index, *TAS* Tegnar activity scale, *K/L* Kellgren–Lawrence grade. A-P and M-L are indicated the tibial tunnel positions in antero-posterior direction (%) and medio-lateral direction (%)

### Longitudinal analysis of modified ACLOAS

Table 2 presents the modified ACLOAS scores before and after surgery. The average interval between the two MRIs was 224.4 (± 138.7) days. Comparing preoperative and postoperative lesion incidence, osteophytes increased significantly in the MTFJ (pre-op: 59.5%; post-op: 83.3%; *p* = 0.02) and LTFJ (pre-op: 30.9%; post-op: 50.0%; *p* = 0.02). However, cartilage lesions did not show any significant increase. Bone marrow lesions significantly improved in the MTFJ (pre-op: 30.9%; post-op: 7.1%; *p* = 0.006) and LTFJ (pre-op: 59.5%; post-op: 7.1%; *p* < 0.001). Additionally, medial meniscal tears significantly decreased (pre-op: 47.6%; post-op: 40.5%; *p* = 0.02), whereas MME significantly increased (pre-op: 30.0%; post-op: 59.5%; *p* < 0.001). No significant changes were observed in lateral meniscal tears or LME.

**Table 2:** OA structural changes detected via MRI in patients who underwent ACL-R

**Modified ACLOAS**										
		MTFJ			LTFJ			PFJ		
		First	Second	*P*	First	Second	*P*	First	Second	*P*
Osteophyte	None	17	7	0.02	29	21	0.02	29	28	1.00
	Grade 1	20	29		11	18		12	14	
	Grade 2	5	6		2	3		1	0	
	Grade 3	0	0		0	0		0	0	
	Frequency	59.5%	83.3%		30.9%	50.0%		30.9%	33.3%	
Cartilage Lesion	None	36	33	0.08	35	36	0.32	37	38	1.00
	Grade 1	6	9		5	4		4	2	
	Grade 2	0	0		2	2		1	2	
	Grade 3	0	0		0	0		0	0	
	Frequency	14.2%	21.4%		16.6%	14.2%		11.9%	9.5%	
Bone marrow lesion	None	29	39	0.006	17	39	<0.001	37	41	0.08
	Grade 1	12	2		14	3		3	1	
	Grade 2	1	1		5	0		2	0	
	Grade 3	0	0		6	0		0	0	
	Frequency	30.9%	7.1%		59.5%	7.1%		11.9%	2.3%	
Meniscus lesion	None	22	25	0.023	22	22	1.00			
	Grade 1	8	12		16	16				
	Grade 2	12	5		4	4				
	Grade 3	0	0		0	0				
	Frequency	47.6%	40.5%		47.6%	47.6%				
Meniscus extrusion	None	25	17	<0.001	33	33	1.00			
	Grade 1	17	23		7	7				
	Grade 2	0	2		2	2				
	Frequency	30.0%	59.5%		21.4%	21.4%				

*MRI* Magnetic resonance imaging, *OA* Osteoarthritis, *ACL-R* Anterior cruciate ligament reconstruction, *ACLOAS* Anterior cruciate ligament osteoarthritis score, *MTFJ* Medial tibiofemoral joint, *LTFJ* Lateral tibiofemoral joint, *PFJ* Patellofemoral joint

### Meniscal extrusion and osteophyte widths

Comparing meniscal extrusion width and osteophyte width before and after ACL-R, MME increased significantly after surgery (first: 1.8 ± 1.1 mm; second: 2.5 ± 1.2 mm; Δ: 0.6 ± 0.6; *p* < 0.001), whereas LME did not show a significant change (first: 1.0 ± 1.1 mm; second: 1.1 ± 1.1 mm; Δ: 0.1 ± 0.8; *p* = 0.32). Osteophyte width significantly increased at all measured sites, including for MFO (first: 1.1 ± 0.8 mm; second: 1.6 ± 0.7 mm; Δ: 0.6 ± 0.6; *p* < 0.001), MTO (first: 1.3 ± 0.8 mm; second: 1.9 ± 1.0 mm; Δ: 0. ± 0.6; *p* < 0.0001), LFO (first: 0.9 ± 0.8 mm; second: 1.1 ± 0.9 mm; Δ: 0.3 ± 0.6; *p* = 0.005), LTO (first: 0.6 ± 0.7 mm; second: 0.8 ± 0.7 mm; Δ: 0.2 ± 0.4; *p* = 0.01) (Table 3). The increase in MME (ΔMME) was significantly correlated with the increase in MTO (ΔMTO) (*r* = 0.67, *p* < 0.001) but not with the increase in MFO (ΔMFO) (*r* = 0.23, *p* = 0.15). The increase in LME (ΔLME) did not correlate with the increase in LFO (ΔLFO) (*r* = 0.05, *p* = 0.76) or the increase in LTO (ΔLTO) (*r* = − 0.11, *p* = 0.47).

**Table 3:** Comparison of meniscus extrusion and osteophyte widths before and after ACL-R surgery

	First	Second	Δ	*p*
MME (mm)	1.8 (1.1)	2.5 (1.2)	0.6 (0.6)	< 0.001
Medial femoral osteophyte width (mm)	1.1 (0.8)	1.6 (0.7)	0.6 (0.6)	< 0.001
Medial tibial osteophyte width (mm)	1.3 (0.8)	1.9 (1.0)	0.5 (0.6)	< 0.001
LME (mm)	1.0 (1.1)	1.1 (1.1)	0.1 (0.8)	0.32
Lateral femoral osteophyte width (mm)	0.9 (0.8)	1.1 (0.9)	0.3 (0.6)	0.005
Lateral tibial osteophyte width (mm)	0.6 (0.7)	0.8 (0.7)	0.2 (0.4)	0.01

Data are presented as means (with standard deviations). Δ, (second)– (first); *ACL-R* Anterior cruciate ligament reconstruction, *MME* Medial meniscus extrusion, *LME* Lateral meniscus extrusion

### Correlation between graft diameter and MME

Graft diameter at the time of surgery was correlated with ΔMME (*r* = 0.48, *p* = 0.001) and ΔMTO (*r* = 0.31, *p* = 0.048). However, there was no significant correlation between graft diameter and ΔMFO (*r* = 0.06, *p* = 0.71), ΔLME (*r* = − 0.02, *p* = 0.88), ΔLFO (*r* = − 0.24, *p* = 0.13), or ΔLTO (*r* = − 0.18, *p* = 0.26). Larger graft diameters were associated with greater progression of MME and MTO (Table 4).

**Table 4:** Meniscal extrusion width and osteophyte width increases in relation to graft diameter

	Graft diameter (mm)								
	7.5 (*n* = 2)	8.0 (*n* = 5)	8.5 (*n* = 4)	9.0 (*n* = 8)	9.5 (*n* = 8)	10 (*n* = 11)	10.5 (*n* = 3)	11 (*n* = 1)	*p*
ΔMME (mm)	0.0 (0.7)	0.1 (0.3)	0.6 (0.6)	0.6 (0.5)	0.7 (0.6)	0.9 (0.6)	0.7 (0.5)	0.6	0.001
ΔMFO (mm)	0.6 (0.6)	0.6 (0.5)	0.4 (0.5)	0.6 (0.6)	0.3 (0.5)	0.9 (0.9)	0.6 (0.1)	0.3	0.71
ΔMTO (mm)	0.2 (0.1)	0.4 (0.4)	0.3 (0.3)	0.4 (0.3)	0.3 (0.5)	0.9 (0.9)	0.6 (0.2)	0.4	0.048
ΔLME (mm)	0.1 (0.1)	0.3 (0.3)	−0.2 (1.3)	0.1 (0.5)	−0.1 (1.1)	0.5 (0.6)	−0.1 (0.2)	−1.0	0.88
ΔLFO (mm)	0.2 (0.4)	0.3 (0.2)	0.4 (0.5)	0.1 (0.2)	0.2 (0.6)	0.4 (0.7)	0.9 (0.9)	1.0	0.13
ΔLTO (mm)	0.4 (0.5)	−0.2 (0.6)	0.4 (0.6)	0.6 (0.3)	0.2 (0.3)	0.4 (0.4)	−0.2 (0.5)	0.7	0.26

Data are presented as means (with standard deviations). *MME* Medial meniscal extrusion, *LME* Lateral meniscal extrusion, *MFO* Medial femoral osteophyte, *MTO* Medial tibial osteophyte, *LFO* Lateral femoral osteophyte, *LTO* Lateral tibial osteophyte

### Comparison between larger and smaller graft diameter groups

**Fig. 2:**
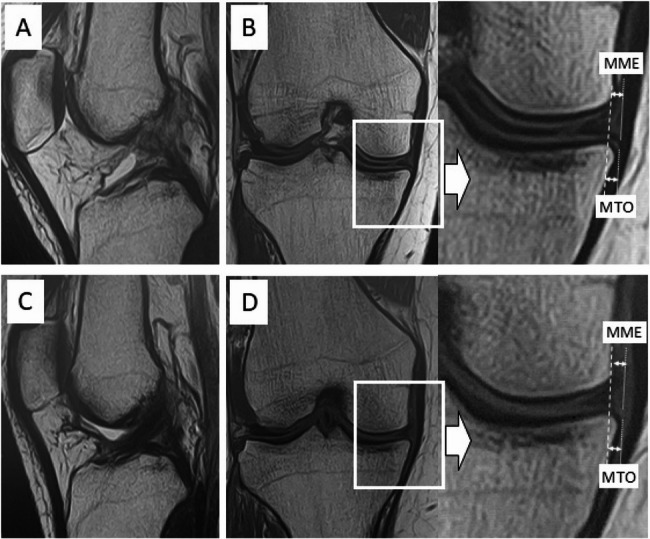
Representative images of a patient from the smaller graft group. Magnetic resonance imaging (MRI) scans before and after anterior cruciate ligament reconstruction (ACL-R) surgery using a 7.5-mm diameter graft in a 28-year-old female professional wrestler. Minimal progression was observed in medial meniscus extrusion (MME) and medial tibial osteophyte (MTO) formation. **A** Preoperative sagittal MRI showing an indistinct ACL. **B** Preoperative coronal MRI showing an MME of 2.5 mm and an MTO of 1.8 mm. **C** Postoperative sagittal MRI image showing the reconstructed ACL graft. **D** Postoperative coronal MRI showing an MME of 2.5 mm and an MTO of 2.1 mm

**Fig. 3:**
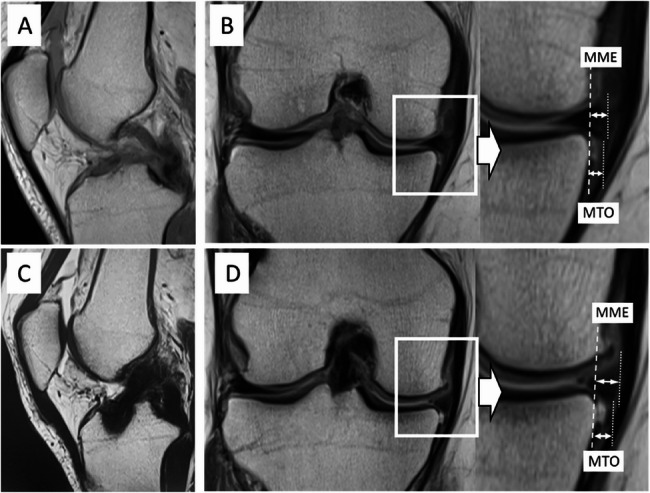
Representative images of a patient from the larger graft group. Magnetic resonance imaging (MRI) scans before and after anterior cruciate ligament reconstruction (ACL-R) surgery using a 9.5-mm diameter graft in a 54-year-old female recreational skier. Noticeable progression in medial meniscus extrusion (MME) width and medial tibial osteophyte (MTO) width was observed, increasing by 1.2 and 1.5 mm, respectively. **A** Preoperative sagittal MRI showing an indistinct ACL. **B** Preoperative coronal MRI showing an MME of 3.3 mm and an MTO of 1.7 mm. **C** Postoperative sagittal MRI showing the reconstructed ACL graft. **D** Postoperative coronal MRI showing an MME of 4.5 mm and an MTO of 3.2 mm

The patients were divided into two groups based on the median graft diameter of 9.25 mm: those with graft diameter greater than 9.25 mm were classified as the larger graft (L) group, and those with graft diameter equal to or less than 9.25 mm as the smaller graft (S) group. The L group exhibited significantly greater ΔMME (L: 0.8 ± 0.6; S: 0.4 ± 0.5; *p* = 0.005) and ΔMTO (L: 0.7 ± 0.7; S: 0.3 ± 0.3; *p* = 0.03). However, ΔMFO (L: 0.6 ± 0.6; S: 0.6 ± 0.5; *p* = 0.005), ΔLME (L: 0.2 ± 0.8; S: 0.1 ± 0.6; *p* = 0.35), ΔLFO (L: 0.4 ± 0.7; S: 0.2 ± 0.3; *p* = 0.13), and ΔLTO (L:0.3 ± 0.4; S: 0.1 ± 0.5; *p* = 0.25) did not differ significantly between the groups (Table 5). Representative images from the S and L groups are shown in Figs. 2 and 3, respectively.

**Table 5:** Patient characteristics and outcomes compared between groups with smaller and larger graft sizes

	Smaller group 7.5–9 mm (*n* = 19)	Larger group 9.5–11 mm (*n* = 23)	*p*
Before ACL-R			
Age	30.1 (9.6)	27.3 (9.0)	0.12
Gender (male/female)	11/8	19/4	0.08
BMI	24.6 (4.5)	25.3 (2.6)	0.28
Tegnar activity scale	7.0 (2.1)	7.4 (1.5)	0.27
K/L grade (0/1/2)	11/7/1	18/5/0	0.62
Femoro-tibial angle (°)	176.6 (2.5)	176.6 (2.3)	0.49
Medial joint space width (mm)	4.0 (0.5)	4.0 (0.3)	0.44
After ACL-R			
Tegnar activity scale	7.0 (2.1)	7.4 (1.5)	0.27
Pivot shift grade (0/1)	14/5	18/5	0.50
K/L grade (0/1/2)	11/6/2	10/9/4	0.26
ΔMME (mm)	0.4 (0.5)	0.8 (0.6)	0.005
ΔMedial femoral osteophyte width (mm)	0.6 (0.5)	0.6 (0.6)	0.45
ΔMedial tibial osteophyte width (mm)	0.3 (0.3)	0.7 (0.7)	0.03
ΔLME (mm)	0.1 (0.6)	0.2 (0.8)	0.35
ΔLateral femoral osteophyte width (mm)	0.2 (0.3)	0.4 (0.7)	0.13
ΔLateral tibial osteophyte width (mm)	0.1 (0.5)	0.3 (0.4)	0.25

Data are presented as means (with standard deviations). Δ, (second)– (first); *ACL-R* Anterior cruciate ligament reconstruction, *BMI* Body mass index, *MME* Medial meniscus extrusion, *LME* Lateral meniscus extrusion, *K/L* Kellgren–Lawrence

## Discussion

In this longitudinal study of patients who underwent ACL-R, osteophyte width and MME significantly increased within 1 year postoperatively, with a greater increase observed in patients with larger graft diameters.

One of the primary goals of ACL-R is to prevent PTOA progression by stabilizing the knee joint and reducing secondary meniscal damage [5]. However, numerous studies have demonstrated that ACL-R does not effectively halt PTOA progression [1, 6, 26], and the mechanisms underlying continued degeneration despite restored stability remain unclear. In the present study, MME progressed postoperatively, despite medial meniscus injuries improving relative to preoperative findings.

If MME develops following ACL-R, it may serve as a risk factor for PTOA progression, suggesting that mitigating its occurrence could help prevent long-term joint degeneration. Previous research has indicated that MME progression coincides with MTO formation [4], which may be attributed to the anatomical connection between the medial meniscus and the medial tibia via the coronary ligament. As osteophytes form on the medial tibia, they may be pushed out of the joint with the attached coronary ligament [15, 27]. The present findings support this notion, as a strong correlation was observed between increases in MME width and MTO width. Conversely, LME progression did not correlate with lateral osteophyte formation, which may be due to the more flexible structure of the lateral meniscus compared with that of the medial meniscus.

**Fig. 4:**
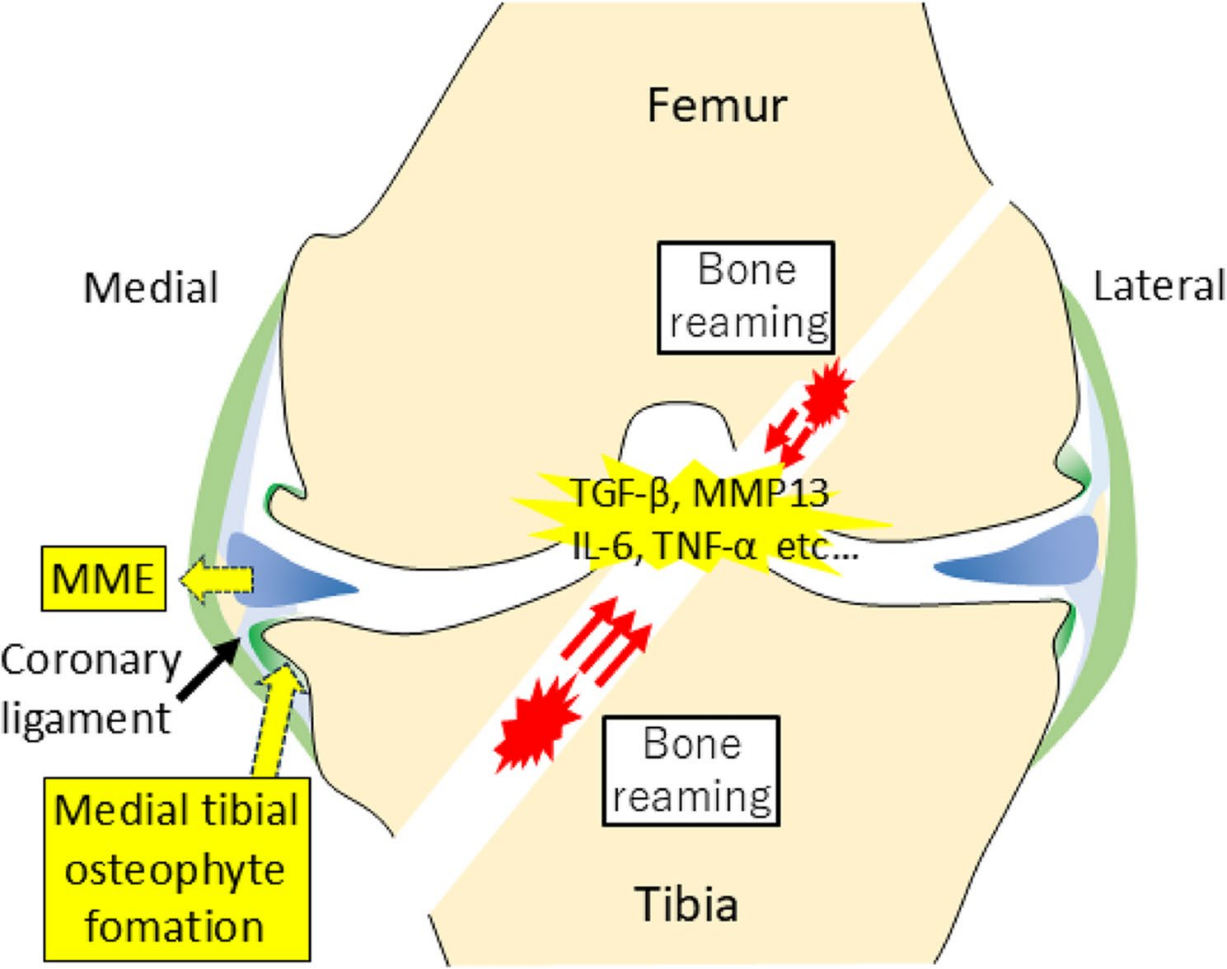
Schematic representation of posttraumatic osteoarthritis progression following anterior cruciate ligament reconstruction surgery. MME, medial meniscal extrusion; TGF-β, transforming growth factor-beta; MMP13, matrix metalloproteinase-13; IL-6, interleukin-6; TNF-α, tumor necrosis factor-alpha

One potential explanation for the greater progression of postoperative MME and MTO widths in patients with larger graft diameters is the increased bone tunnel size required for graft insertion, with larger tunnels leading to more damage, including greater intra-articular bone destruction. ACL-R is known to induce an increase in proinflammatory cytokine levels, including those of interleukin-6 and tumor necrosis factor-alpha, which contribute to knee OA progression, along with intra-articular hematoma and inflammation [17]. Additionally, drilling into the joint space during bone tunnel creation promotes the release of MMP3 and TGF-β, both of which have been implicated in osteophyte formation [13, 14, 18]. The medial tibial bone tunnel is typically the largest, making it particularly susceptible to these effects, which may explain why MTO progression correlated with graft diameter more strongly compared with osteophyte changes in other regions (Fig. 4). In addition to the potential biological effects, it is also conceivable that a larger bone tunnel diameter may impose increased mechanical stress on the anterior root attachment of the medial meniscus. This stress could contribute to the progression of medial meniscal extrusion [28], particularly in cases where the anterior root integrity is compromised or subjected to altered joint mechanics following ACL-R.

To reduce the risk of PTOA following ACL-R, it may be important to consider strategies for minimizing osteophyte formation and MME progression.

While a few studies have reported that combining ACL-R with meniscal extrusion-focused procedures may help suppress extrusion [29], the clinical efficacy and long-term benefit of such approaches remain controversial. In contrast, several longitudinal MRI studies, including the present study, have demonstrated progression of meniscal extrusion following standard ACL-R with or without conventional meniscal repair [30]. These findings suggest that meniscal extrusion may persist or worsen despite restored joint stability. Further investigation is needed to clarify whether targeted interventions for meniscal extrusion can meaningfully alter postoperative joint mechanics or the risk of PTOA. We acknowledge that non-anatomical tunnel placement could contribute to postoperative joint degeneration. However, in our cohort, the tunnel positions were generally anatomical, and no correlation was observed between tunnel placement and the progression of meniscal extrusion. Notably, the progression of MME following ACL-R has been consistently reported in the literature [4, 11, 30] implying that intrinsic joint degeneration or biomechanical factors may also play a significant role.

This study’s findings suggest that graft diameter, and consequently bone tunnel size, play a role in degenerative changes and should be considered during surgical planning. However, minimizing rerupture risk after ACL-R is also a critical consideration, with studies showing that a graft diameter ≤ 8 mm is associated with a higher risk of rerupture, whereas larger grafts reduce this risk [31]. Notably, excessive graft size has been linked to postoperative complications, such as roof impingement leading to abrasions, collagen fiber damage, and delayed blood vessel reconstruction. Blood flow restoration following ACL-R begins at the graft periphery owing to contributions from the Hoffa fat pad and synovium, but overly large grafts may lead to prolonged hypovascularity in the graft center, limiting blood flow for several weeks postoperatively [32–34]. Additionally, although patellar tendon and quadriceps tendon grafts offer increased strength and reduced rerupture rates, they are directly connected to the knee joint and have been associated with a higher risk of OA progression compared with hamstring grafts, which provide extra-articular support [35]. Thus, graft selection should be individualized based on activity level, rerupture risk, OA progression risk, and patient-specific needs, The present study suggests that graft diameter also warrants consideration in this decision-making process.

Optimal grafts with strength and a small diameter may require biological augmentations aimed at enhancing ligament maturation and mechanical strength. In particular, platelet-rich plasma therapy has shown promise in promoting ligament maturation and preventing retears [36, 37]. Additionally, techniques that avoid or minimize bone tunnel drilling should be explored [38].

Despite ongoing debate regarding ACL-R failing to prevent PTOA, to the best of our knowledge, this study is the first to demonstrate a relationship between graft diameter and PTOA progression, and the study’s findings could provide clinical insights into how graft diameter affects OA progression. Nonetheless, this study has several limitations. First, MME measurements were based on MRI scans taken in the supine position, meaning that the findings may underestimate results for axially loaded knees. Therefore, future studies incorporating weight-bearing MRI and ultrasonography could provide more accurate assessments. Second, the analysis was limited to short-term postoperative changes, making it unclear how the findings translate to long-term PTOA progression. Third, as a retrospective study, the results are subject to selection bias. Therefore, a randomized, longitudinal study with a larger sample size is required to validate these results and reduce potential confounding factors. Fourth, the timing of postoperative MRI acquisition was not standardized across patients. Although this reflects real-world clinical practice, such variability may have influenced the degree of detectable PTOA-related changes. Future studies should consider fixed or serial imaging intervals to better capture the temporal dynamics of postoperative joint degeneration.

## Conclusion

Within 1 year after ACL-R, MME increased in correlation with MTO formation, with greater progression observed in patients with larger graft diameters.

## Data Availability

The datasets used and/or analysed during the current study are available from the corresponding author on reasonable request.

## Abbreviations

OA: Osteoarthritis
PTOA: Posttraumatic osteoarthritis
ACL: Anterior cruciate ligament
MRI: Magnetic resonace imaging
MME: Medial meniscal extrusion
ACL-R: Anterior cruciate ligament reconstruction
BMI: Body mass index
TAS: Tegnar activity scale
K/L: Kellgren–Lawrence grade
ACLOAS: Anterior cruciate ligament osteoarthritis score
MTFJ: Medial tibiofemoral joint
LTFJ: Lateral tibiofemoral joint
PFJ: Patellofemoral joint
LME: Lateral meniscal extrusion
MFO: Medial femoral osteophyte
MTO: Medial tibial osteophyte
LFO: Lateral femoral osteophyte
LTO: Lateral tibial osteophyte
TGF-β: Transforming growth factor-beta
MMP13: Matrix metalloproteinase-13
IL-6: Interleukin-6
TNF-α: Tumor necrosis factor-alpha
